# Bavachin Suppresses Alpha-Hemolysin Expression and Protects Mice from Pneumonia Infection by *Staphylococcus aureus*

**DOI:** 10.4014/jmb.2207.07048

**Published:** 2022-09-14

**Authors:** Ye Tao, Dazhong Sun, Xinran Ren, Yicheng Zhao, Hengjian Zhang, Tao Jiang, Jiyu Guan, Yong Tang, Wu Song, Shuqiang Li, Li Wang

**Affiliations:** 1 Changchun University of Chinese Medicine, Changchun 130117, P.R. China; 2 Center for Pathogen Biology and Infectious Diseases, Key Laboratory of Organ Regeneration and Transplantation of the Ministry of Education, The First Hospital of Jilin University, State Key Laboratory of Human-Animal Zoonotic infectious Diseases, Changchun, P.R. China; 3 The Affiliated Hospital to Changchun University of Chinese Medicine, Changchun, P.R. China; 4 First School of Clinical Medicine, Guangzhou University of Chinese Medicine, Guangzhou 510006, P.R. China; 5 School of Pharmaceutical Science, Jilin University, Changchun 130021, P.R. China; 6 Key Laboratory of Zoonosis, Ministry of Education, College of Veterinary Medicine, Jilin University, Changchun 130062, P.R. China; 7 Department of Orthopedic Surgery, The First Hospital of Jilin University, Changchun 130062, P.R. China

**Keywords:** Bavachin, *Staphylococcus aureus*, alpha-hemolysin, pneumonia

## Abstract

*Staphylococcus aureus* (*S. aureus*) infection causes dramatic harm to human health as well as to livestock development. As an important virulence factor, alpha-hemolysin (hla) is critical in the process of *S. aureus* infection. In this report, we found that bavachin, a natural flavonoid, not only efficiently inhibited the hemolytic activity of hla, but was also capable of inhibiting it on transcriptional and translational levels. Moreover, further data revealed that bavachin had no neutralizing activity on hla, which did not affect the formation of hla heptamers and exhibited no effects on the hla thermal stability. In vitro assays showed that bavachin was able to reduce the *S. aureus*-induced damage of A549 cells. Thus, bavachin repressed the lethality of pneumonia infection, lung bacterial load and lung tissue inflammation in mice, providing potent protection to mice models in vivo. Our results indicated that bavachin has the potential for development as a candidate hla inhibitor against *S. aureus*.

## Introduction

*Staphylococcus aureus* (*S. aureus*) is a gram-positive opportunistic pathogenic bacterium. In livestock farming, mastitis caused by *S. aureus* infection in cows results in decreased milk production, decreased milk quality, and increased cow mortality. As such, infections caused by *S. aureus* inflict significant economic losses on the farming industry [1]. In addition, other animals such as pigs, chickens, cats, and dogs also carry *S. aureus*, which can induce superficial skin diseases, otitis externa, pneumonia, and sepsis, imposing serious harm on animal health [2-4]. The carriage rate of *S. aureus* is about 20% in dairy cattle and as high as 90% in chickens [5]. Meanwhile, as *S. aureus* can be transmitted from animals to humans, *S. aureus* infection poses a serious challenge to public health safety and livestock development [2].

In recent years, high selection pressure on antibiotics has allowed the rapid spread of resistant strains [6]. Among them, methicillin-resistant *Staphylococcus aureus* (MRSA) is a highly virulent and drug-resistant pathogen. Known as a multi-drug resistant “superbug,” MRSA has been reported to be resistant to numerous antibiotics. The prevalence of MRSA in livestock animals has also accelerated the emergence of antibiotic resistance, making it more difficult to treat mastitis, especially in dairy cows [7]. Moreover, the mortality rate after MRSA infection is three times higher than that of common *S. aureus*, and the resulting high cost and difficulty of treatment has become a worldwide public health problem [8-10].

At present, vancomycin, daptomycin, and linezolid are preferred agents against MRSA infection in clinical settings [10]. However, certain MRSA strains have developed mild resistance to vancomycin (MIC ≥ 16 μg/ml), and the development of new potent antibiotics is fraught with challenges [10, 11]. Therefore, there is an urgent need to develop new alternative strategies to combat *S. aureus* infections, including anti-virulence therapy, photodynamic therapy, phage therapy, vaccines, nanoparticle therapy, etc. [8, 10]. In addition, combination therapeutic strategies are also effective against *S. aureus* infections and have been applied in several fields [12, 13]. Such strategies feature a different mechanism of action than conventional antibiotics, with the main objective of the anti-virulence approach being inhibition of virulence factor production with few inhibitory proliferation effects from *S. aureus*. Anti-virulence treatment does not affect the host flora and generates less selective pressure, acting on non-bacterial pathways and thus delaying the emergence of drug resistance [14, 15].

*S. aureus* produces numerous virulence factors that are required to infect host cells. Alpha-hemolysin (Hla) [16] is a water-soluble, β-barrel pore-forming toxin encoded by the *hla* gene and is usually secreted in large amounts during the later phases of *S. aureus* growth [8]. Secreted as a monomer (33.2 kDa), hla self-assembles on the host cell membrane and is capable of forming a transmembrane heptamer (232.4 kDa), resulting in host cell lysis and death [17]. Hla is toxic to many mammalian cells, including erythrocytes, platelets, epithelial cells, and lymphocytes. Rabbit red blood cells (RBCs) are extremely sensitive to hla, 1,000 times more sensitive than human RBCs [18]. In addition, hla has been shown to have cutaneous necrotic and neurotoxic properties, which are a major virulence factor for *S. aureus* to evade the host immune system. A study showed that the pathogenicity of *S. aureus* was significantly diminished after knocking out hla [19]. Hence, it is crucial to target hla in studies against *S. aureus* infections.

Natural products have become the focus of current research for novel drugs due to their multiple pharmacological functions [20]. In this study, to screen the active ingredients that inhibit the virulence factors of important pathogenic bacteria, hemolysis analysis was used to screen hla inhibitors from hundreds of traditional Chinese medicine monomers in a library of natural small-molecule compounds that have been constructed in the laboratory. At present, several highly active hla inhibitors have been obtained, including bavachin. Bavachin (also named coryfolin) is the main flavonoid extracted from the fruits of *Psoralea corylifolia* Linn [21, 22]. We found that bavachin effectively inhibited the transcription and translation levels of hla and had a protective effect against A549 cell infection caused by *S. aureus*. Moreover, it was able to significantly reduce the infection in a mice pneumonia infection model in vivo. Bavachin could be applied to *S. aureus* and has strong application prospects.

## Materials and Methods

### General Study Design

First, MIC and growth curve measurements of bavachin against *S. aureus* USA300 were carried out. The inhibitory effect of bavachin on the hemolytic effect of *S. aureus* USA300 was investigated, as well as its inhibitory effect on the hemolytic activity of *S. aureus* Newman and the clinical isolate SA1B3G. The effect of bavachin on the transcription and protein levels of *hla* in *S. aureus* USA300 was examined by western blot and RT-qPCR, as well as its effect on the transcription levels of virulence factors *agrA* and *RNAIII*.

Next, the effect of bavachin on hla activity was investigated. The effect of bavachin on hla heptamer formation was examined by oligomerization assay and neutralization activity assay, and whether bavachin binds to hla protein was examined by CETSA.

Last, the effect of bavachin on *S. aureus* infection was studied in vitro and in vivo. We examined the effect of bavachin in A549 cell infection by *S. aureus* and constructed a mouse pneumonia infection model to detect the effect of bavachin on *S. aureus*-infected mice.

### Strains, Cells, and Drugs

*S. aureus* USA300 (ATCC BAA-1717) and Newman (ATCC 8325) were purchased from American Type Culture Collection (ATCC, USA). Clinical isolates of MRSA strain SA1B3G were isolated in Shenyang (Liaoning, China) and identified in the isolation unit for quality control. They were stored in 20% glycerol-rich BHI at -80°C and passaged twice on tryptic soy agar before the experiment. *E. coli* BL21-pET28a-hla was previously constructed in the laboratory. Trypticase Soy Broth (TSB) and Luria-Bertani (LB) were used for the general culture of *S. aureus* and *E. coli*, respectively, at 37°C and 200 rpm. A549 cells were cultured in Roswell Park Memorial Institute-1640 (RPMI-1640) medium containing 10% FBS and penicillin-streptomycin, 5% CO_2_, at 37°C. Bavachin (purity ≥ 98%, additional file 1) was purchased from Chengdu Herbpurify Co., Ltd. (China), dissolved in DMSO to a final concentration of 10 mg/ml, and stored at −20°C. Bavachin is a dioxyflavonoid with a molecular mass of 324.37, molecular formula C_20_H_20_O_4_, and the chemical structure formula is (*S*)-2,3-dihydro-7-hydroxy-2-(4-hydroxyphenyl)-6-(3-methyl-2-butenyl)-4*H*-1-benzopyran-4-one.

### Measurement of Minimum Inhibitory Concentration (MIC) and Growth Curves

The MIC of bavachin against *S. aureus* USA300 was determined using the broth microdilution method according to Clinical and Laboratory Standards Institute (CLSI) guidelines [23]. *S. aureus* USA300 was incubated in TSB until the OD_600_ value was 0.3, which was used to determine the growth curve. This was followed by the addition of DMSO or 16 μg/ml of bavachin, respectively. *S. aureus* remained in the culture at 37°C, 180 ×*g*, and the OD_600_ value was measured every hour during the exponential growth period, and every 2 h or longer during the late exponential growth period.

### Hemolysis Assay

To detect the inhibitory effect of bavachin on the hemolysis of *S. aureus* USA300, Newman and SA1B3G were treated with different concentrations of bavachin (2, 4, 8, 16 μg/ml) overnight, respectively. After centrifugation at 5,000 ×*g* for 5 min, 100 μl of supernatant was mixed with 875 μl PBS and 25 μl rabbit RBCs and incubated at 37°C for 30 min. Triton X-100 was used as the positive control and PBS as the negative control. The mixture was centrifuged at 4,000 ×*g* and 4°C for 3 min. Hemolysis was observed and the mixture was transferred to a 96-well plate; the absorbance value at OD_543_ nm was measured by a microplate reader.

To detect the neutralizing activity of bavachin on hla, the supernatant of an overnight culture of USA300 was centrifuged and incubated with different concentrations of bavachin (8, 16, 32, 64 μg/ml) at 37°C for 30 min, then PBS and rabbit RBCs were added for another 30 min. The absorbance values were also measured as above.

### Western Blot

To measure the effect of bavachin on hla protein expression, the USA300 supernatant treated with different concentrations of bavachin in the hemolysis assay was mixed with a 5× protein loading buffer. Samples were boiled at 100°C for 5 min followed by a 12% SDS-PAGE analysis. Then, the proteins were electrotransferred to polyvinylidene fluoride (PVDF) membranes, followed by blocking of the membranes for 2 h with 5% skim milk (Beyotime, China). Membranes were incubated with Anti-*Staphylococcal* α-Toxin antibody (diluted 1:5,000) (Cat. No. S7531) overnight at 4°C. The binding of the primary antibody was detected by HRP conjugated goat anti-rabbit antibody (diluted 1:2, 000). Blots were detected using the ECL detection system (Bio-Rad, USA).

### RT-qPCR

The effect of bavachin on transcript levels of *hla*, *RNAIII*, and *agrA* was examined by RT-qPCR. After treatment with different concentrations of bavachin or DMSO overnight at 37°C, with shaking until the OD_600_ reached 2.5, total RNA was extracted by the TRIzol method. The BeyoRT II First Strand cDNA Synthesis Kit (Beyotime) was used for cDNA synthesis. qPCR was performed using NovoStart SYBR qPCR SuperMix Plus (Novoprotein, China). 16sRNA was used as the reference gene and the primer sequences used were as follows: 16S forward primer 5’-TGATCCTGGCTCAGGATGA-3’, 16S reverse primer 5’-TTCGCTCGACTTGCATGTA-3’; *hla* forward primer 5’-GGTATATGGCAATCAACTT-3’, *hla* reverse primer 5’-CTCGTTCGTATATTACATCTAT-3’; *RNAIII* forward primer 5’-AATTAGCAAGTGAGTAACATTTGCTAGT-3’, *RNAIII* reverse primer 5’-GATGTTGTTTACGATAGCTTACATGC-3’; *agrA* forward primer 5’-GCAGTAATTCAGTGTATGTTCA-3’, *agrA* reverse primer 5’-TATGGCGATTGACGACAA-3’. The expression of genes was obtained by calculating 2^-ΔΔCt^ after normalizing the cycle threshold (Ct) of each gene with the Ct value of the housekeeping gene (16S RNA).

### Purification of Recombinant Hla Protein

*E. coli* BL21-pET28a-hla was cultured in LB to an OD_600_ of 0.6, and Isopropyl-beta-D-thiogalactopyranoside IPTG at a final concentration of 1.0 mM was added and incubated overnight at 16°C for substantial induction of protein production. The bacteria were resuspended in a binding buffer for ultrasonic fragmentation, and the protein was purified by BeyoGold His-tag Purification Resin (Beyotime). Hla was purified in 50 mM and 100 mM imidazole elution buffer.

### Oligomerization Assay

The effect of bavachin on hla heptamer formation was detected by an oligomerization assay. The purified hla (0.5 mg/ml final concentration) was added with different concentrations of bavachin (8, 16, 32, 64, 128 μg/ml) or DMSO and then incubated with sodium deoxycholate (5 mM final concentration) at 22°C for 30 min to induce heptamer formation in vitro. Then, the mixture was added to 5× protein loading buffer without β-mercaptoethanol and incubated for 10 min at 55°C. Samples were electrophoresed through 8% SDS-PAGE. Gels were stained with Coomassie brilliant blue to visualize protein bands.

### Cellular Thermal Shift Assay (CETSA)

CETSA was used to detect whether bavachin works on hla protein. Meanwhile, *E. coli* BL21-pET28a-hla was cultured and induced to express hla protein. The supernatant after the sonication above was incubated in 64 μg/ml bavachin or DMSO at 37°C for 1 h and centrifuged at 18,000 g for 20 min. The supernatants were heated at different temperatures (40-60°C) for 5 min, centrifuged again at 18,000 ×*g* for 20 min. The supernatants were used as protein samples for 12% SDS-PAGE. The gels were stained with Coomassie brilliant blue and observed in a gel imaging system (Bio-Rad).

### Cytotoxicity Assay

Bavachin toxicity to A549 cells was detected by methylthiazolyldiphenyl-tetrazolium bromide (MTT) assays [24].

### A549 Cell Infection Assay

A549 cells were plated in 24-well plates with 2 × 10^4^ cells per well. When the cells adhered, *S. aureus* USA300 was added for infection. *S. aureus* USA300 was prepared by resuspension in 1640 medium after growth to an OD_600_ value of 0.8. The non-infected group, the DMSO group, and the bavachin-treated group with different concentrations were designed respectively. Infection was carried out at 37°C for 3 h. After infection, each group of cultures was collected and lactate dehydrogenase (LDH) [25] was measured according to the manufacturer's instructions. Meanwhile, cells were stained with live/dead staining, and the numbers of surviving and dead cells were observed under an inverted fluorescence microscope.

### Mouse Pneumonia Model

Pneumonia models were constructed using 6-to-8-week-old female C57BL/6J mice. For the lethality assays, mice were anesthetized and inhaled 2 × 10^8^ CFU/ml *S. aureus* USA300 through the nasal cavity. Mice in the treatment group (*n* = 10) were injected subcutaneously each 12 h with 40 mg/kg bavachin (0.1 ml) or an equal volume of saline in the control group. The survival of mice was observed after infection at 12 h intervals for a total of 72 h.

Mice were infected with non-lethal doses of *S. aureus* USA300 (1 × 10^8^ CFU/ml) for pathological observation. Lung tissues from USA300-infected and bavachin-treated mice were ground and serially diluted in Tryptone Soya Agar (TSA) plates for colony counting. In parallel, the lungs were photographed and the lung tissues were stained with hematoxylin and eosin (H&E) and photographed with a microscope.

### Data Statistics and Analysis

Data were obtained from three parallel measurements and presented as mean ± SD. Data were analyzed by the two-tailed Student’s *t*-test or one-way ANOVA for normal distribution, and others were analyzed by the two-tailed Mann-Whitney U test for abnormal distribution. *p* < 0.05 was statistically significant.

## Results

### Bavachin Does Not Inhibit *S. aureus* Growth and Has No Obvious Cytotoxicity

**Fig. 1:**
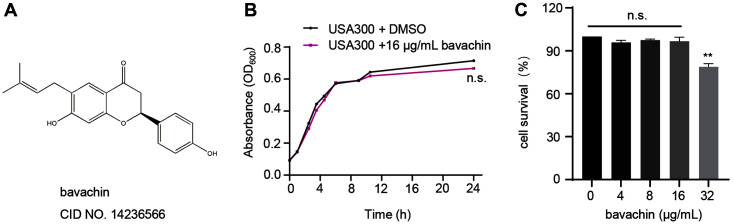
Effect of bavachin on *S. aureus* and cells. (**A**) Chemical structure of bavachin. (**B**) Growth curves of *S. aureus* USA300 with or without the addition of 16 μg/ml bavachin. (**C**) Measuring the toxic effects of different bavachin concentrations (4, 8, 16, 32 μg/ml) on A549 cells by MTT assay. **** *p* < 0.0001, *** *p* < 0.001, ** *p* < 0.01, * *p* < 0.05.

First, we measured the MIC value of bavachin at 32 μg/ml against *S. aureus* USA300 (Fig. 1A). Then, the concentration of bavachin at 1/2 MIC value was chosen for determining the growth curve. The results showed that bavachin at 16 μg/ml did not inhibit the growth of *S. aureus* USA300 (Fig. 1B). Meanwhile, MTT assays showed that there was no significant cytotoxicity of bavachin on A549 cells at 16 μg/ml (Fig. 1C).

### Bavachin Inhibits the Hemolytic Activity of *S. aureus*

**Fig. 2:**
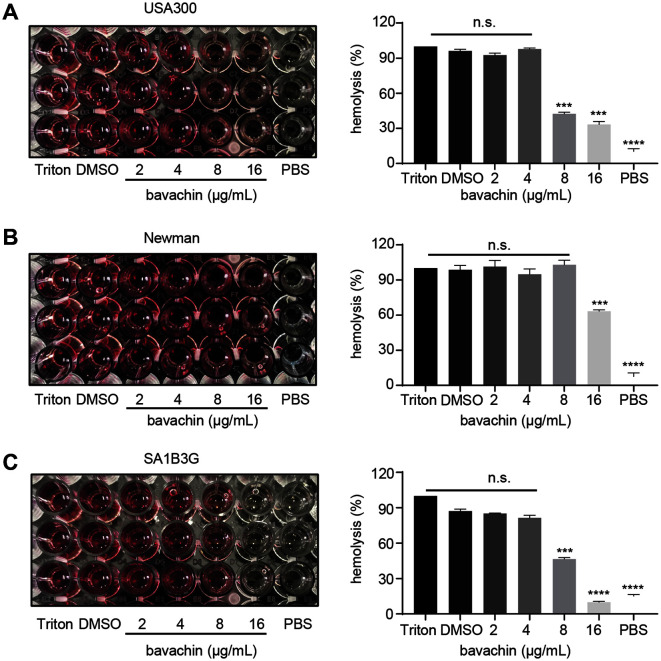
The inhibitory effect of bavachin on the hemolysis of *S. aureus*. The hemolytic inhibitory effect of *S. aureus* USA300 (**A**), Newman (**B**), and SA1B3G (**C**) was detected by hemolysis assay after treatment with different concentrations of bavachin (2, 4, 8, 16 μg/ml). **** *p* < 0.0001, *** *p* < 0.001, ** *p* < 0.01, * *p* < 0.05.

Hemolytic assays were performed and the results indicated that bavachin inhibited the hemolytic activity of *S. aureus* USA300 in a dose-dependent manner (Fig. 2A). Bavachin at 16 μg/ml inhibited the hemolytic activity of *S. aureus* USA300 by more than 80%. In addition, we also detected the effect of bavachin on *S. aureus* Newman and the clinical isolate *S. aureus* SA1B3G. The results demonstrated that bavachin had an inhibitory effect on the hemolytic activity of both Newman (Fig. 2B) and SA1B3G (Fig. 2C).

### Bavachin Does Not Directly Interact with Hla Protein.

**Fig. 3:**
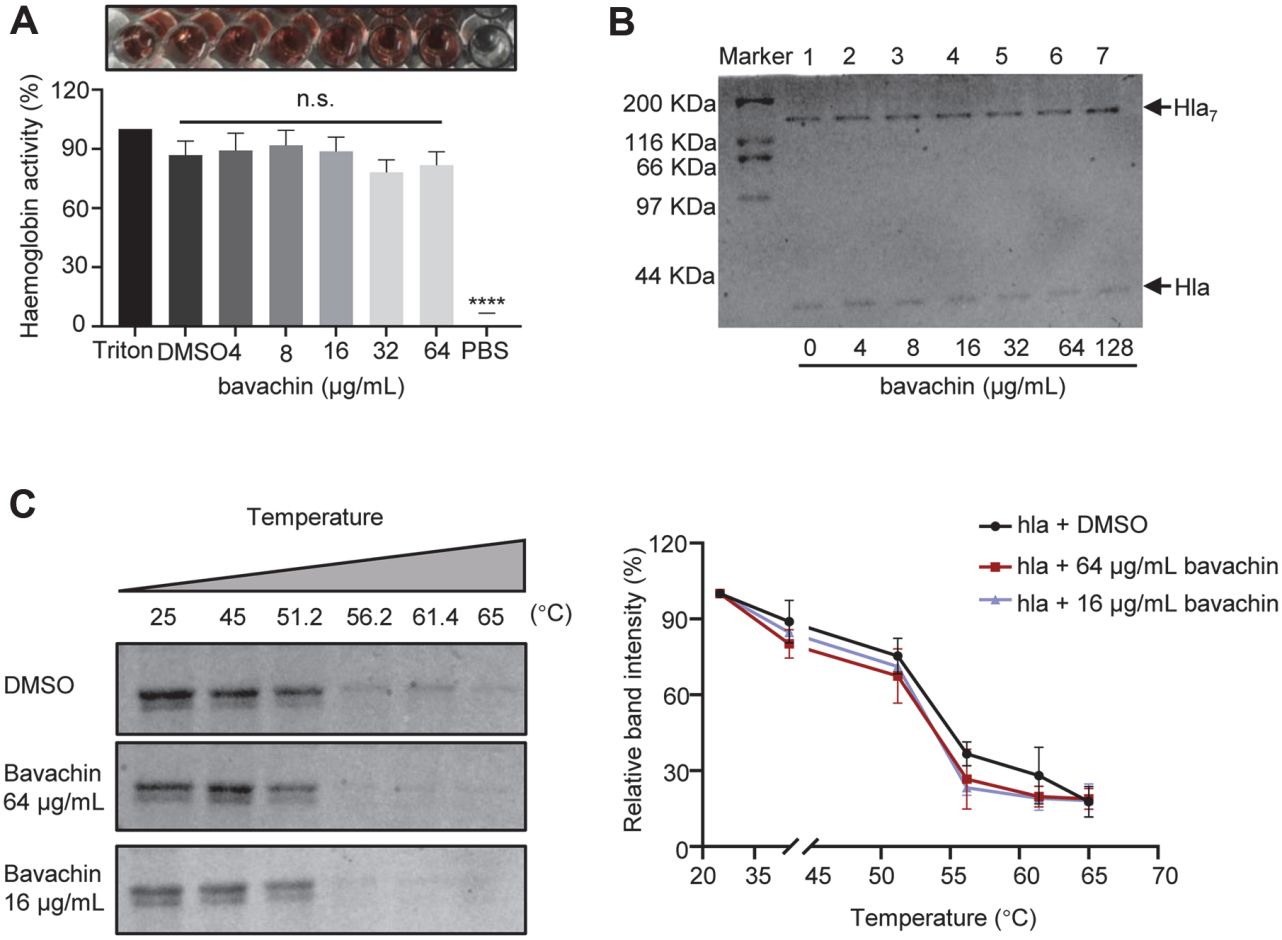
Effect of bavachin on hla protein. (**A**) The neutralizing activity of different concentrations of bavachin (4, 8, 16, 32, 64 μg/ml) against hla in *S. aureus* USA300 supernatant was detected by neutralization assays. (**B**) Oligomerization assays were used to detect the effect of bavachin (4, 8, 16, 32, 64, 128 μg/ml) on the heptamer formation of hla proteins in vitro. (**C**) The CETSA assays examined the effect of bavachin (16 and 64 μg/ml) on the thermal stabilization of hla after different temperatures treating hla, and the protein bands at room temperature (25°C) were used as 100% for statistical analysis.

First, we tested whether bavachin has a neutralizing effect on hla by neutralization assays. As shown in Fig. 3A, bavachin did not affect the hemolytic activity of hla in the supernatant of *S. aureus*, indicating no neutralizing activity against hla. The formation of heptamers is essential for the pathogenicity of hla, and it was revealed by oligomerization assays that bavachin did not inhibit hla heptamers even at a concentration of 128 μg/ml (Fig. 3B). Simultaneously, the CETSA assays showed that bavachin did not affect the thermal stability of hla protein (Fig. 3C). In conclusion, the inhibitory effect of bavachin on *S. aureus* hemolysis was not directly produced by binding to hla protein.

### Bavachin Represses the Expression of Hla and Transcription of Hla-Related Virulence Genes

**Fig. 4:**
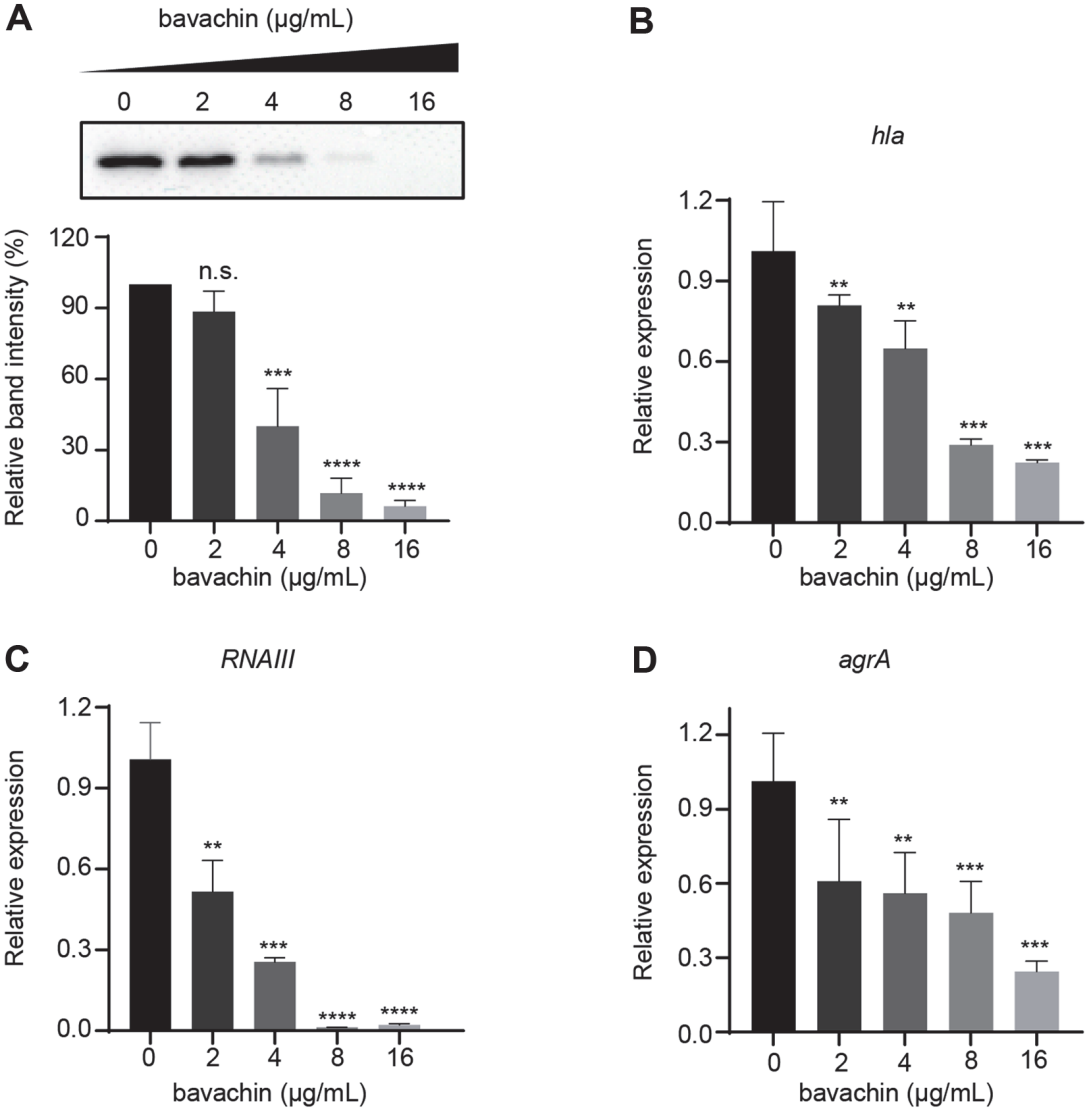
The effect of bavachin on the transcriptional and translational levels of hla in *S. aureus*. (**A**) The expression level of hla in the supernatant of *S. aureus* USA300 with or without bavachin (2, 4, 8, 16 μg/ml) was observed by western blot. The effect of bavachin on the transcript levels of hla (**B**), *agrA* (**C**), and *RNAIII* (**D**) was examined by RT-qPCR. **** *p* < 0.0001, ***p < 0.001, **p < 0.01, *p < 0.05.

The influence of bavachin on hla expression was detected by western blotting and the results were consistent with hemolysis assay data. Bavachin suppressed the expression of *S. aureus* hla in a dose-dependent manner (Fig. 4A), and almost the entire hla expression could be suppressed at 16 μg/ml (Fig. 4A). The performance of RT-qPCR also showed that bavachin significantly repressed the transcript levels of *hla* (Fig. 4B). In addition, we examined the effect of bavachin on the upstream genes of *hla*. As seen in Figs. 4C-4D, bavachin also repressed the transcript levels of *agrA* and *RNAIII*, which were crucial virulence genes in *S. aureus*.

### Protective Effect of Bavachin on A549 Cells

**Fig. 5:**
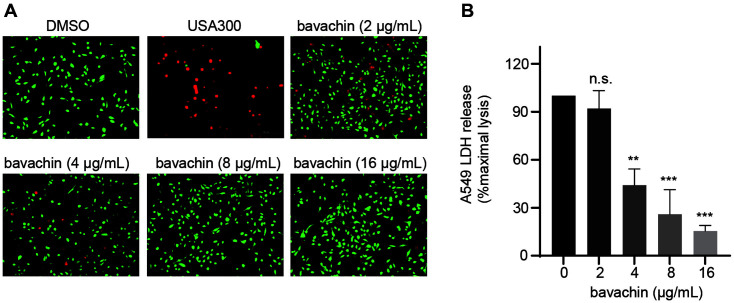
Effect of bavachin on A549 cells infected with *S. aureus*. (**A**) A549 cells were stained with live/dead staining after *S. aureus* USA300 infection. Live cells were colored green and dead cells were colored red. Each group of cells was treated with DMSO (uninfected), *S. aureus* USA300, and *S. aureus* USA300 added with different bavachin concentrations (2, 4, 8, 16 μg/ml), respectively. (**B**) The post-infection supernatant in (**A**) was collected and the LDH release in each well was measured, with the DMSO group serving as a negative control and the *S. aureus* USA300-infected group as a positive control.

To visualize the effect of bavachin on A549 cells infected with *S. aureus*, Calcein AM/PI staining was performed on the infected cells. The results indicated that there was a large quantity of cell death after infection with *S. aureus* USA300. In contrast, the addition of bavachin treatment significantly improved cell mortality and attenuated the infection of A549 by *S. aureus* (Fig. 5A). The assays showed that cell death following *S. aureus* infection resulted in a dramatic increase in the release of LDH, and bavachin, acting as a protective agent for A549 cells, effectively reduced this LDH release (Fig. 5B).

### Bavachin Prevents Pneumonia Infection in Mice

**Fig. 6:**
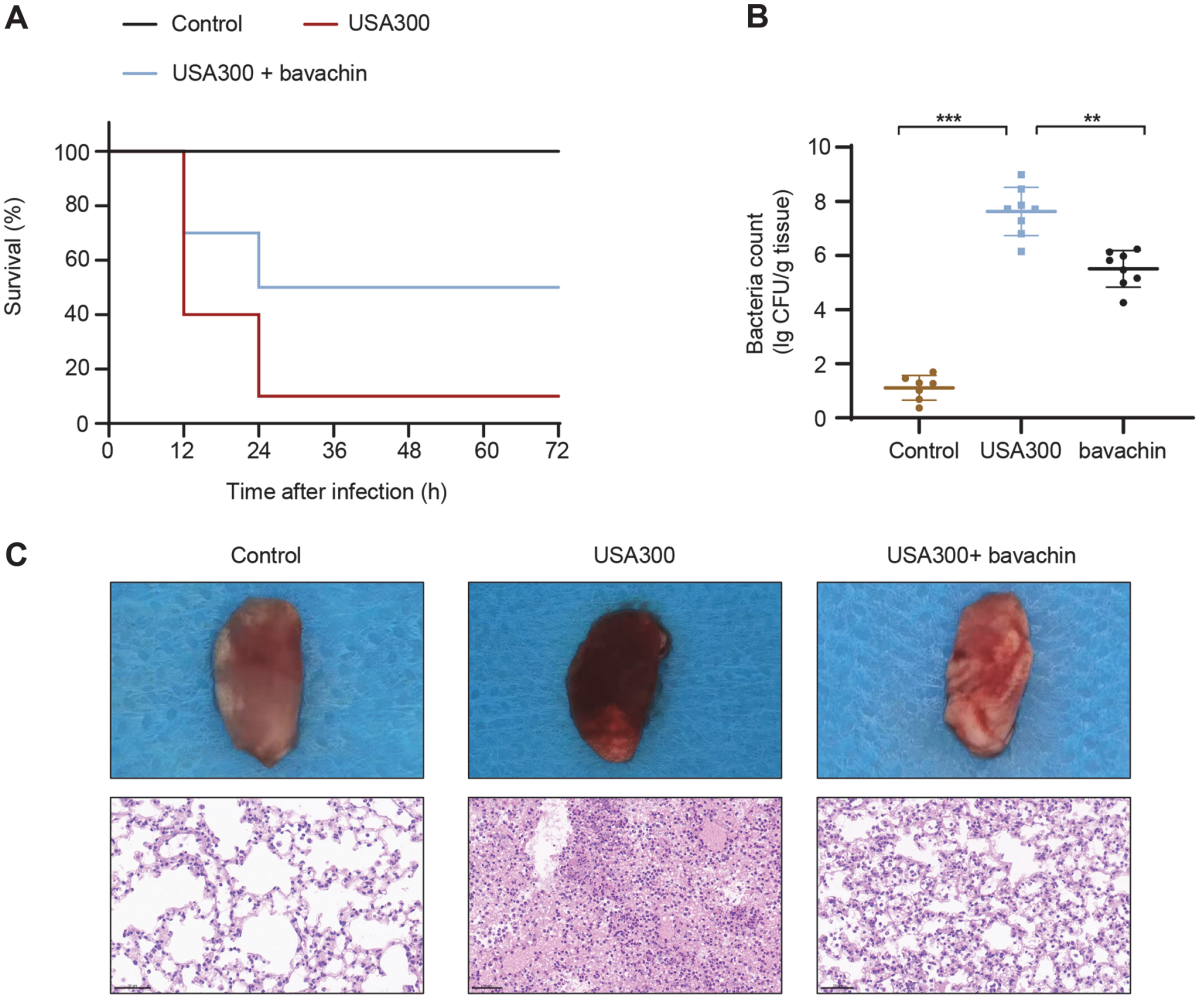
The effect of bavachin on pneumonia infection in mice. (**A**) After the mice (*n* = 10) were intranasally infected with 2 × 10^8^ CFU/ml of *S. aureus* USA300, the mortality rate of each group was recorded within 72 h. After the mice were infected with non-lethal doses (1 × 10^8^ CFU/ml) of *S. aureus* USA300, the bacterial load (**B**) of lung tissues in the infected and bavachin-treated groups was measured, (**C**) and the lung tissues were observed by H&E staining. Scale bar, 50 μm.

A mouse pneumonia infection model was constructed to observe the effect of bavachin in *S. aureus*-infected mice. In the lethality assays, mice administered nasally with 2 × 10^8^ CFU/ml *S. aureus* had a mortality rate of 60%within 12 h, and the cumulative mortality rate increased to 90% within 24 h. After the treatment with a subcutaneous injection of 40 mg/kg of bavachin, the mortality rate was significantly reduced, and showed a cumulative mortality rate of 50% at 24 h (Fig. 6A), to the extent that the lethal infection of mice by *S. aureus* was mitigated. In a non-lethal model, mice were treated with 1 × 10^8^ CFU/ml *S. aureus* by intranasal inhalation and the bavachin was also administered. The mice were dissected after 48 h of treatment and the lung tissues of control and drug-treated mice were counted for colonies. The statistical results showed that the lung tissue load of mice with *S. aureus* infection was 7.62 ± 0.83 log_10_ CFU/g, and the CFU of mice in the treated group was significantly decreased (5.51 ± 0.63 log_10_ CFU/g) (Fig. 6B). Lung tissue from mice infected with *S. aureus* was inspected to assess the protective effect of bavachin. H&E staining of lung tissue revealed almost no infiltrative inflammatory cells in the control mice. However, there was significant inflammatory cell infiltration in the lung tissue of *S. aureus* infected mice. Importantly, the addition of bavachin significantly attenuated lung injury and a reduction in infiltrative inflammatory cells was observed. A similar protective effect of bavachin against pneumonia infection in mice was also observed in lung appearance photographs (Fig. 6C).

## Discussion

Infamous for its high virulence and antibiotic resistance, MRSA has become a stumbling block to clinical treatment and currently represents a serious public health challenge worldwide [10]. Therefore, new additional therapeutic strategies are urgently needed to treat infections caused by *S. aureus*. Moving forward, there is increasing concern about targeting regulatory pathways as a tool to prevent infections [8]. Among these, hla disrupts eukaryotic cell membrane integrity through the receptor ADAM10, induces apoptosis and cell lysis, and participates in the inflammatory response, making it a nasty pore-forming toxin [26, 27]. For these reasons, hla has become a research hotspot as one of the most important virulence factors involved in the pathogenicity of *S. aureus* [28].

The hla inhibitor bavachin identified in the studies has been reported to possess a variety of pharmacological activities, including immunomodulatory, antitumor, antioxidant, antibacterial, antifungal, anti-inflammatory, antiviral, and estrogenic activities [21, 22, 29, 30]. Bavachin effectively repressed hla production and hemolytic activity without affecting the growth of *S. aureus*. At 16 μg/mL, bavachin was capable of significantly inhibiting the hemolytic activity of MRSA USA300 and MSSA Newman. Most importantly, it exhibited significant inhibitory hemolytic activity against clinical isolates, but we also found that the MIC value of bavachin was only 32 μg/ml. We observed that 32 μg/ml of bavachin would inhibit the growth of *S. aureus* to some extent when the growth curve was measured.

Related studies have also shown that extracts of *P. corylifolia* had antibacterial activity [31]. Therefore, it is probable that bavachin at different doses has multiple roles in anti-virulence and antibacterial activity against *S. aureus*. This study focused on the inhibitory effect of bavachin on the virulence factor of *S. aureus* in the dose range that did not affect the growth of *S. aureus*.

In this work, we found that flavonoids in a variety of natural products [32], such as red wine [33], quercetin and tannic acid [34], could effectively inhibit the infection ability of *S. aureus*, and significantly inhibited the biofilm formation and hemolytic activity of *S. aureus*. Bavachin, which we investigated in this study, is also a flavonoid and was effective in inhibiting the hemolytic activity of *S. aureus*. Therefore, we hypothesized whether the mechanism by which flavonoids could inhibit the infection of *S. aureus* was related to its specific chemical structure, which we would like to investigate next.

Hla heptamer formation is essential for the pathogenicity of *S. aureus* USA300. Studies have shown that hla mutation results in heptamers with no hemolytic activity, which could be used as an immune vaccine against hla [35]. In addition, the P103C and N105C mutations significantly reduced hla hemolysis and heptamer formation [36]. Whereas our study found that bavachin inhibited the transcriptional and translational processes, it reduced the secretion of hla in *S. aureus*, but did not affect hla heptamer formation and did not directly interreact with hla protein. This suggested that bavachin could potentially target other parts of the virulence factor regulatory network in *S. aureus*. To this end, we evaluated additionally the effect of bavachin on transcript levels of *agrA* and *RNAIII*. Hla protein is the pathogenic virulence factor of *S. aureus*, and its expression is closely related to the quorum sensing-assisted gene regulation (*agr*) system [37]. In the study of hla protein mechanisms, the agr gene has two transcripts, called *RNAII* and *RNAIII* [38]. *RNAII* is the operon of four genes, *agrB*, *agrD*, *agrC* and *agrA*, which encode important peptides and proteins in the quorum sensing system. AgrD forms autoinducing peptides (AIPs) under the action of AgrB protein, and AIPs are phosphorylated by binding to AgrC receptor [39]. Phosphorylated AgrC activates AgrA and phosphorylates it. Phosphorylated AgrA can bind to the P3 promoter and drive *RNAIII* transcription [40]. *RNAIII* is an important virulence factor in *S. aureus*. It can upregulate the toxin genes associated with infection, including *hla*, *pvl* and *spa*. As bavachin also affected the transcriptional levels of *agrA* and *RNAIII*, the implication is that bavachin influenced hla by affecting the agr sensing system potentially. The deeper mechanism of action still needs to be further investigated. A series of hla inhibitors have been reported to protect A549 cells from hla-mediated injury, and consistent results were observed in our assays [17, 41]. In addition, bavachin attenuated the lethality of pneumonia infection in mice and reduced lung tissue inflammation, effectively protecting against *S. aureus*-induced pneumonia infections in vivo and providing a valid basis for developing bavachin as an anti-*S. aureus* virulence inhibitor.

In summary, this work developed a potent inhibitor that effectively downregulated the expression level of hla. Furthermore, bavachin significantly alleviated the pneumonia infection in mice caused by *S. aureus* and thereby demonstrated the excellent application potential of this flavonoid. Our data provided new options for the prevention of *S. aureus* infections such as mastitis in animals and can be expected to help reduce economic loss to the livestock industry and also to lay the groundwork for research into the prevention and treatment of *S. aureus* infections.
